# Molecular Cloning and Functional Characterization of a Dihydroflavonol 4-Reductase from *Vitis bellula*

**DOI:** 10.3390/molecules23040861

**Published:** 2018-04-10

**Authors:** Yue Zhu, Qingzhong Peng, Kegang Li, De-Yu Xie

**Affiliations:** 1 Hunan Provincial Key Laboratory of Plant Resources Conservation and Utilization, College of Biology and Environmental Sciences, Jishou University, No. 120 Ren Min Nan Lu, Jishou City 416000, China; yzhu31@ncsu.edu (Y.Z.); lkg@jsu.edu.cn (K.L.); 2 Department of Plant Biology, North Carolina State University, 100 Derieux Place, Raleigh, NC 27695, USA

**Keywords:** *Vitis bellula*, dihydroflavonol reductase, dihydroflavonol, leucoanthocyanidin, anthocyanin, proanthocyanidin

## Abstract

*Vitis bellula* is a new grape crop in southern China. Berries of this species are rich in antioxidative anthocyanins and proanthocyanidins. This study reports cloning and functional characterization of a cDNA encoding a *V. bellula* dihydroflavonol reductase (VbDFR) involved in the biosynthesis of anthocyanins and proanthocyanidins. A cDNA including 1014 bp was cloned from young leaves and its open reading frame (ORF) was deduced encoding 337 amino acids, highly similar to *V. vinifera* DFR (VvDFR). Green florescence protein fusion and confocal microscopy analysis determined the cytosolic localization of VbDFR in plant cells. A soluble recombinant VbDFR was induced and purified from *E. coli* for enzyme assay. In the presence of NADPH, the recombinant enzyme catalyzed dihydrokaempferol (DHK) and dihydroquercetin (DHQ) to their corresponding leucoanthocyanidins. The VbDFR cDNA was introduced into tobacco plants via *Agrobacterium*-mediated transformation. The overexpression of VbDFR increased anthocyanin production in flowers. Anthocyanin hydrolysis and chromatographic analysis revealed that transgenic flowers produced pelargonidin and delphinidin, which were not detected in control flowers. These data demonstrated that the overexpression of VbDFR produced new tobacco anthocyanidins. In summary, all data demonstrate that VbDFR is a useful gene to provide three types of substrates for metabolic engineering of anthocyanins and proanthocyanidins in grape crops and other crops.

## 1. Introduction

**Figure 1:**
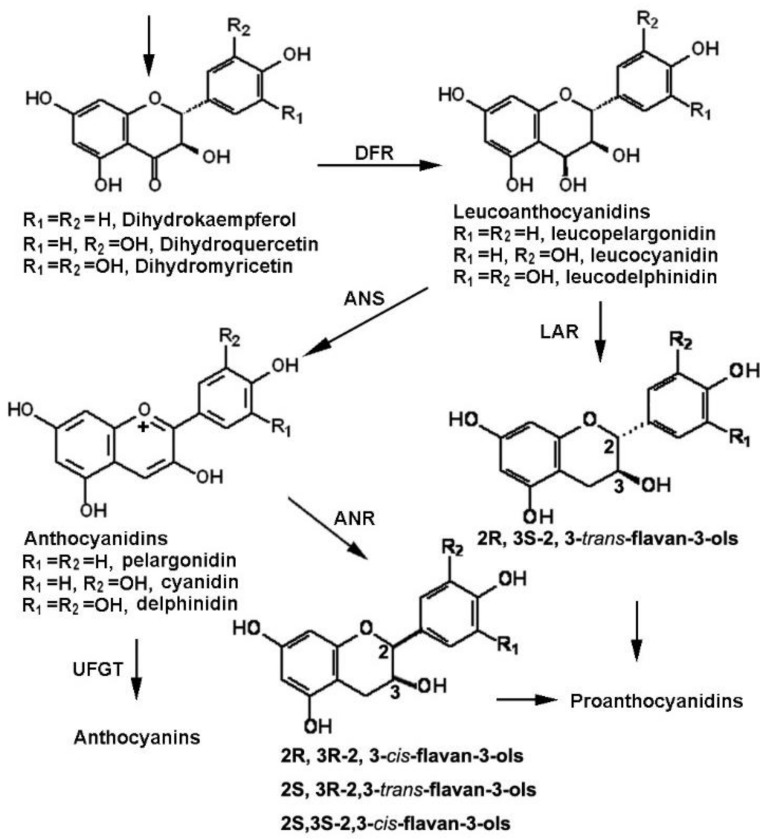
Late pathway steps of flavonoids from dihydroflavonol to anthocyanins and proanthocyanidins. DFR: dihydroflavonol reductase, ANS: anthocyanidin synthase, ANR: anthocyanidin reductase, LAR: leucoanthocyanidin reductase, and UFGT: UDP-glucose flavonoid 3-*O*-glucosyltransferase. UDP: uridine diphosphate.

Dihydroflavonol 4-reductase (DFR) is a key late enzyme in the plant flavonoid pathway toward both anthocyanins and proanthocyanidins (Figure 1). It catalyzes the key step from dihydroflavonols, such as dihydrokaempferol (DHK), dihydroquercetin (DHQ), and dihydromyricetin (DHM), to leucoanthocyanidins, such as leucopelargonidin, leucocyanidin, and leucodelphinidin [1]. DFR genes have been cloned from multiple plants and its mutation has been demonstrated to cause the loss of anthocyanins and proanthocyanidins in plants [2,3,4,5]. To date, *DFR* is an economically important plant gene, given that anthocyanins and proanthocyanidins are two groups of antioxidants relating to high nutritional values of crop, food, and beverage (such as wine and green tea) products [6,7,8,9]. Particularly, anthocyanins are one of the richest plant natural pigments with significant economic values in the horticulture industry [6,10,11,12,13]. Since the first success of *Petunia* flower color engineering was achieved using the first DFR cDNA cloned from maize [13], multiple homologs have been cloned to engineer anthocyanins [1,14,15] and proanthocyanidins [16,17]. DFR also plays a key role in transcription factor-based anthocyanin engineering. A common metabolic phenotype is that the activation or enhancement of *DFR* expression is necessary in MYB transcription factor-based anthocyanin engineering. For example, the regulatory function of the Production of Anthocyanin Pigmentation 1 (PAP1, a MYB75) depends upon the expression of *DFR* [18,19,20,21,22,23,24].

*Vitis bellula* (namely Bellula here) is a relative of *V. vinifera* (in Vitaceae), which is the main grape crop for both wine and non-alcoholic beverage products [25,26,27]. Although the cropping of *V. bellula* is limited to southern China [28], this species has become an emerging new grape crop to develop potential wine and beverage products due to its high nutritional values. Our recent studies have revealed that its berry is rich in flavan-3-ols and proanthocyanidins [28,29] (Figure 1). We have demonstrated that the formation of proanthocyanidins and flavan-3-ols in *V. bellula* is via two pathways, the anthocyanidin reductase (ANR) and leucoanthocyanidin reductase (LAR) pathways [28,30]. Metabolic profiling revealed that Bellula’s berries produce two types of configurations of flavan-3-ols, 2R, 3S-2, 3-*trans*-flavan-3-ols such as (+)-catechin and (+)-gallocatechin and 2R, 3R-2, 3-*cis*-flavan-3-ols such as (−)-epicatechin (and (−)-epigallocatechin. Both butanol:HCl cleavage and chromatograph analyses have revealed that the building units of proanthocyanidins include three types of flavan-3-ols, which are characterized by one –OH, two –OH, and three –OH groups in the B ring, such as (−)-epifazelechin, (−)-epicatechin, and (−)-epigallocatechin (Figure 1). These three types of structures are correspondingly derived from leucopelargonidin, leucocyanidin, and leucodelphinidin via either the ANR or LAR pathway (Figure 1).

To date, cDNA(s) encoding DFR has not be cloned from *V. bellula*. In this article, we report the cloning and characterization of *V. bellula* DFR (VbDFR). A DFR cDNA homolog, namely VbDFR, was cloned from young leaves. Sequence alignment using deduced amino acids showed that only three amino acids were different between VbDFR and *V. vinifera* DFR (VvDFR), demonstrating their high identity. In vitro enzymatic assay, transgenic analysis, and metabolic profiling of transgenic plants showed that VbDFR catalyzes the step from dihydroflavonols to leucoanthocyanidins. Particularly, the overexpression of VbDFR in tobacco flowers led to the formation of pelargonidin, cyanidin, and delphinidin in plants. These results indicate that VvDFR is a useful gene to metabolically engineer three types of anthocyanidins and their corresponding anthocyanins.

## 2. Results

### 2.1. Cloning of DFR Gene from Leaf of V. bellula

**Figure 2:**
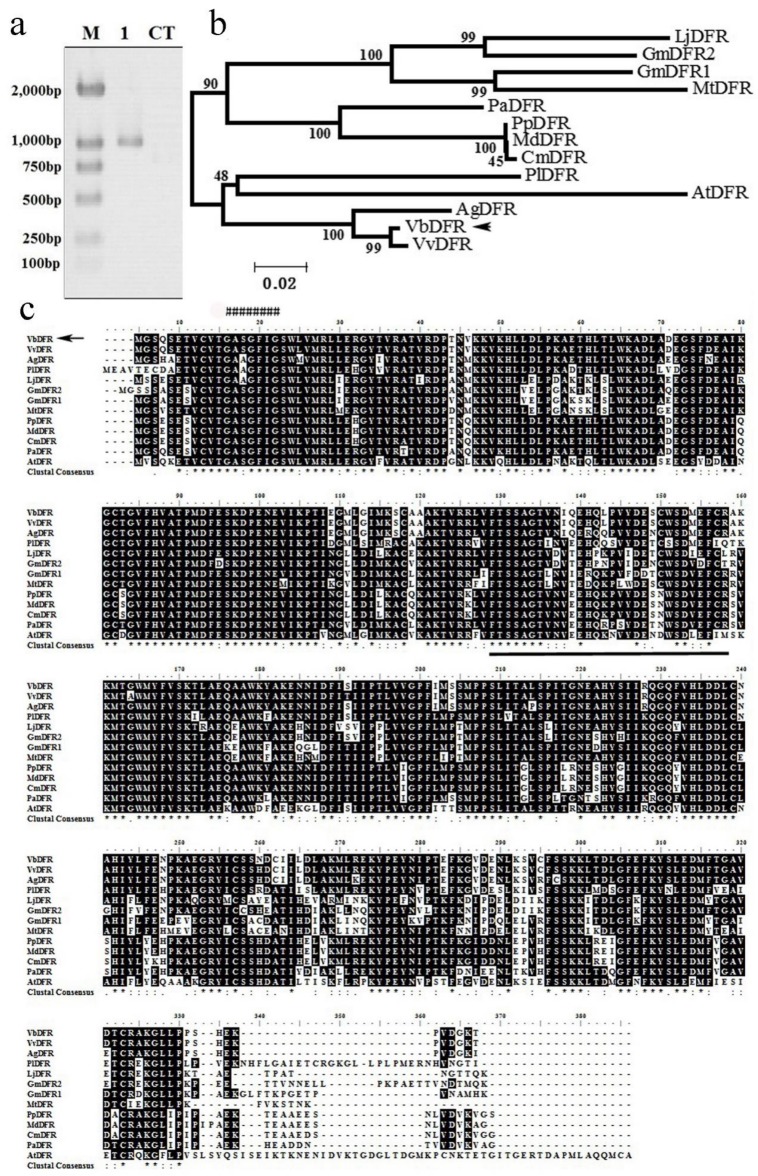
Cloning, amino acid sequence alignment, and an unrooted phylogenetic tree of DFR obtained from deduced amino acid sequences of 13 DFR homologs. (**a**) a *DFR* cDNA fragment was amplified from *V. bellula* leaf tissue by RT-PCR. M: DNA marker, 1: *DFR* cDNA fragment, CT: negative control; (**b**) an unrooted phylogenetic tree was built from amino acid sequences of 13 DFR homologs; (**c**) amino acid sequence alignment were develop from 13 DFR homologs; “*”: the same amino acid in all sequences; “:”: conserved amino acid residues; “.”: half conserved amino acid residues; “######”: potential NADPH/NADH binding domain; amino acids underlined form a potential substrate specificity domain of DFR. AtDFR: *Arabidopsis thaliana* DFR; CmDFR: *Crataegus monogyna* DFR; GmDFR1: *Glycine max* DFR1; GmDFR2: *Glycine max* DFR2; LjDFR: *Lotus japonicas* DFR; MdDFR: *Malus domestica* DFR; MtDFR: *Medicago truncatula* DFR; NgDFR: *Nekemias grossedentata* DFR; PaDFR: *Prunus avium* DFR; PpDFR: *Pyrus pyrifolia* DFR; PlDFR: *Paeonia lactiflora* DFR; VvDFR: *Vitis vinifera* DFR.

Based on VvDFR sequence (GenBank: CAA53578.1), we designed a pair of primers, which contained the start and stop codon nucleotides. RT-PCR was carried out to amplify a DFR cDNA from a leaf cDNA library of *V. bellula*. We named it VbDFR. This amplification produced an approximately 1 kb cDNA fragment (Figure 2a). Sequencing confirmed that this fragment was composed of 1014 nucleotides including the full length of open reading frame (ORF) from the start codon to the stop codon, which was deduced to translate 337 amino acids. An unrooted phylogenetic tree was built using amino acid sequences of 13 DFR homologs. The resulting tree clustered them into two main clades (Figure 2b). VbDFR, VvDFR, AtDFR, AgDFR, and PlDFR were clustered in one clade, which VbDFR and VvDFR are directly clustered together, indicating their close similarity. Further amino acid sequence alignment using 13 DFR homologs revealed three different amino acids between VbDFR and *V. vinifera* DFR (VvDFR). In addition, this alignment showed that the deduced VbDFR amino acid sequence included the conserved glycine-rich Rossmann NADPH/NADH-binding domain and a substrate specificity domain (Figure 2c).

### 2.2. Recombinant VbDFR Expression and Enzymatic Assay 

**Figure 3:**
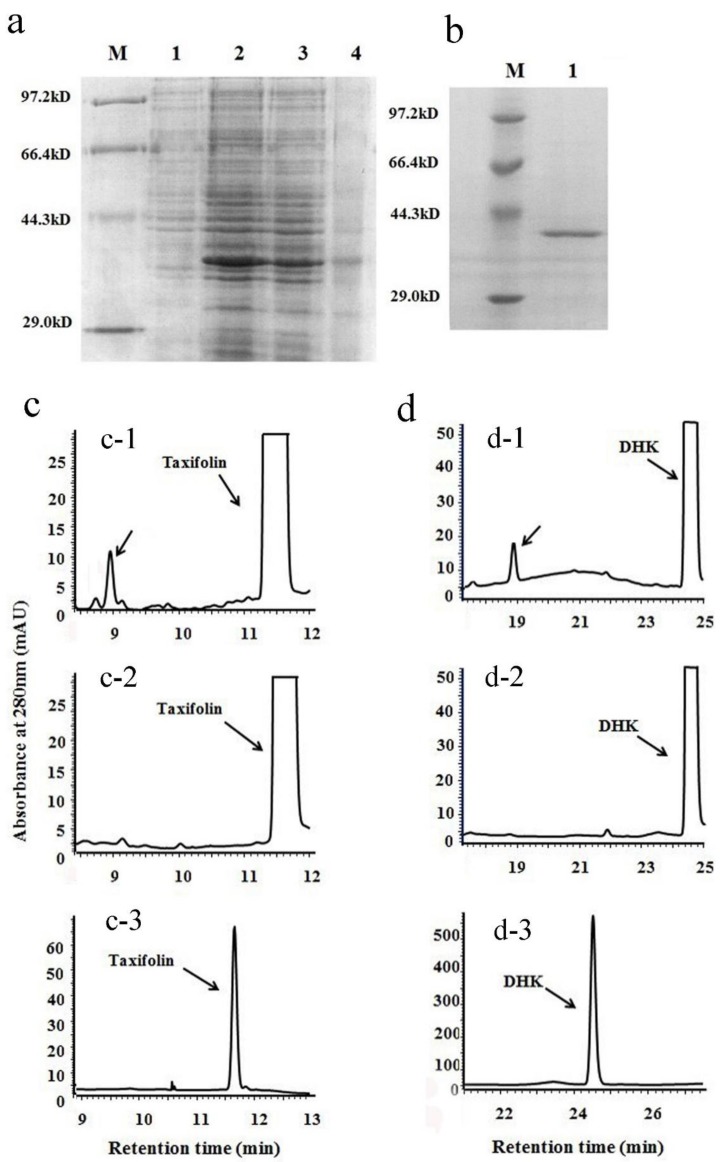
Recombinant protein expression and enzymatic analysis of VbDFR. (**a**) a SDS-PAGE image shows recombinant VbDFR protein induced in *E. coli* BL21 (DE3) plysS strain. Lane 1: 20 μg crude protein extracts from BL21 (DE3) plysS/pET28a (+) vector control; lane 2: 20 μg crude protein extracts from BL21 (DE3) plysS/pET28a (+)-VbDFR. Lane 3: insoluble crude protein extracts from BL21 (DE3) plysS/pET28a (+)-VbDFR. Lane 4: soluble crude protein extracts from BL21 (DE3) plysS/pET28a (+)-VbDFR. M: protein molecular weight marker; (**b**) an image shows recombinant VbDFR purified; (**c**) HPLC profiles show one product formed from the incubation of taxifolin and recombinant VbDFR (c-1) but not denatured VbDFR (c-2); c-3, taxifolin standard; (**d**) HPLC profiles show one product formed from the incubation of dihydrokaempferol (DHK) and recombinant VbDFR (d-1) but not denatured recombinant VbDFR (d-2); d-3 DHK standard.

The ORF was cloned into a pET 28a (+) vector to obtain a recombinant pET 28a (+)-VbDFR vector. This construct was transformed into BL21 (DE3) plysS strain to induce recombinant VbDFR protein. The pET 28a (+) empty vector was used as control. After induced with 1.0 mM IPTG, extraction of *E. coli* revealed that the recombinant VbDFR protein existed on both inclusion body and buffer (Figure 3a). However, the recombinant protein was not produced from the empty vector control (Figure 3a). Next, the recombinant protein was purified by using a Ni-NTA column. SDS-PAGE analysis showed one major recombinant VbDFR band from this purification (Figure 3b). The purified recombinant VbDFR was stored at −20 °C for further enzymatic assays. (−)-Taxifolin and (−)-dihydrokaempferol (DHK) are two DFR substrates (Figure 1). We used these two substrates to examine the enzymatic activity of the recombinant VbDFR. The enzymatic reaction was carried out in 500 μL volume that included 15 μg substrates, 50 μg protein, 50 mM citrate buffer at pH 6, and 20 μg NADPH. After reactions were extracted with ethyl acetate, products were dissolved in methanol for HPLC analysis. HPLC profiles recorded at 280 nm showed that one new peak was observed from reactions using taxifolin (Figure 3c-1) and DHK (Figure 3d-1), respectively. However, these new peaks were neither observed from control reactions using denatured protein nor existed in standard samples (Figure 3c-2,d-2). These results demonstrated that the recombinant VbDFR used these two metabolites as substrates. Although leucoanthocyanidin standards were not commercially available, based on our previous elucidation method for DFR’s products [1], these two new peaks from taxifolin and DHK were annotated to be leucocyanidin and leucopelargonidin, respectively.

### 2.3. Subcellular Localization of VbDFR

**Figure 4:**
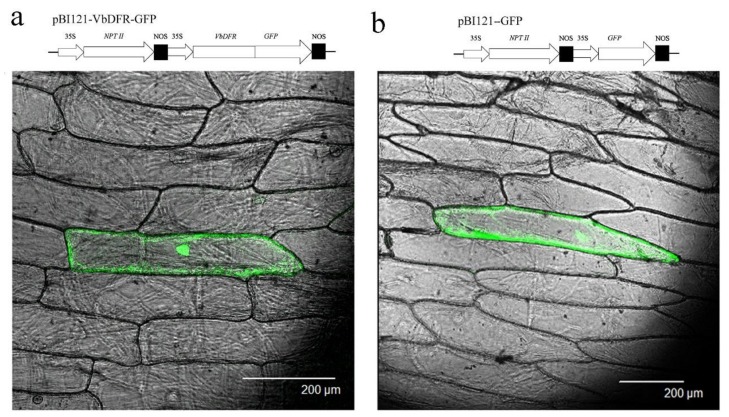
Transient expression using VbDFR-GFP fusion and confocal microscope images. (**a**) a cassette showing VbDFR and GFP fusion and a confocal microscopy image showing subcellular localization of VbDFR-GFP; (**b**) a cassette showing GFP as positive control and a confocal microscopy image showing subcellular localization of GFP.

After the stop codon was removed, *VbDFR* was fused to the 5-terminus of green fluorescent protein (GFP) to obtain a new plasmid, namely pBI121-VbDFR-GFP (Figure 4a). In addition, a pBI121-GFP plasmid was used as control. Two types of plasmids were introduced to onion epidermal cells via a gene gun transformation. Examination of epidermal cells under confocal microscope revealed that green fluorescence signal was mainly located in the entire cytosol of epidermal cells transformed with the pBI121-VbDFR-GFP plasmid (Figure 4a). The same result was observed in epidermal cells transformed with the pBI121-GFP plasmid (Figure 4b). These results showed the cytosolic localization of VbDFR.

### 2.4. Overexpression of VbDFR Tobacco Enhances Production of Anthocyanins

**Figure 5:**
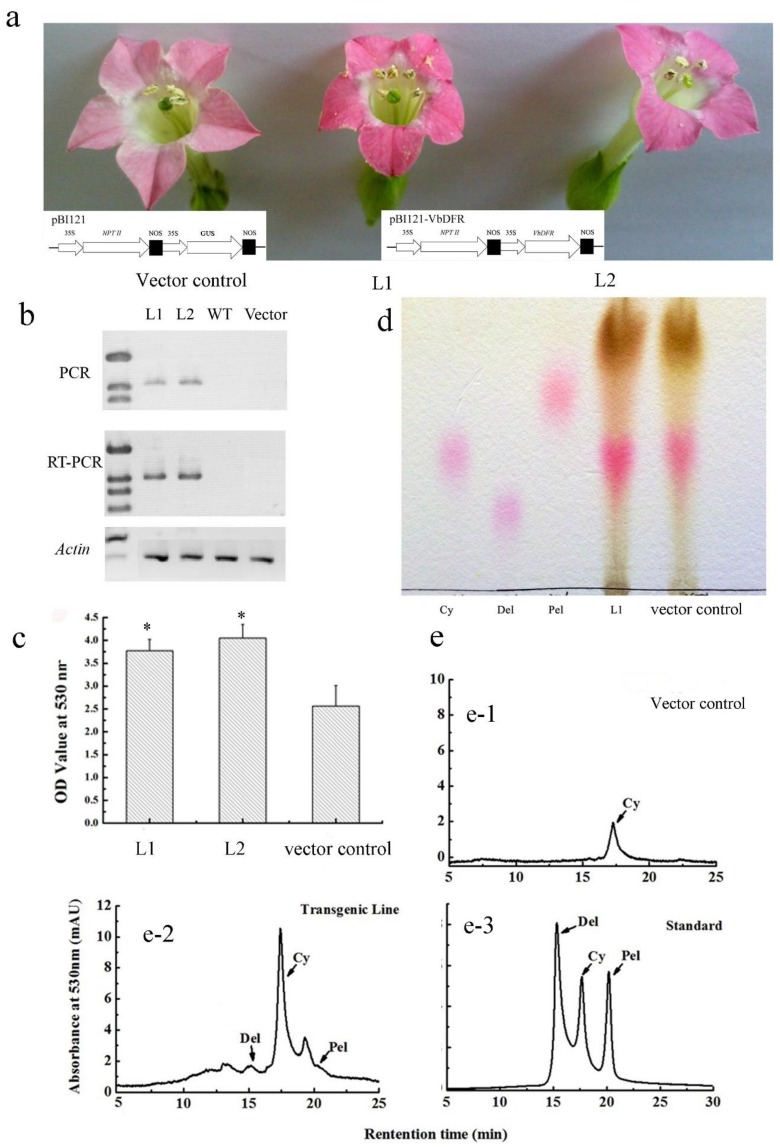
Increase of anthocyanins in transgenic tobacco flowers overexpressing *VbDFR*. (**a**) cassettes of pBI121 and pBI121-VbDFR vectors show *VbDFR* controlled by 35S promoter for overexpression, and red pigmentation was enhanced in flowers of two *VbDFR* transgenic tobacco lines (L1 and L2) compared with vector control flower; (**b**) images show fragments amplified by genomic DNA-based PCR and RT-PCR; (**c**) absorbance values for anthocyanin extracts were significantly higher from flowers of *VbDFR* transgenic tobacco lines (L1 and L2) than from vector control flowers; (**d**) TLC profiles show anthocyanidins from the butanol:HCl hydrolysis of anthocyanins extracts from transgenic tobacco line (L1) and vector control plants; (**e**) HPLC profiles show anthocyanidins from butanol:HCl hydrolysis of anthocyanins extracted from transgenic tobacco line (e-1) and vector control (e-2) plants. e-3: authorized standards, Del: delphinidin, Cy: cyanidin, Pel: pelargonidin. * significant difference between transgenic and wild-type control flowers.

VbDFR was overexpressed in tobacco plants to examine its function *in planta*. The ORF of VbDFR was cloned into the pBI121 binary vector under the control of a CaMV35S promoter (Figure 5a) and transformed to tobacco plants as reported previously [1]. More than 10 kanamycin-resistant plantlets were regenerated from tissue culture, planted in pot soil, and maintained in the greenhouse to grow up for flowers. In addition, pBI121 vector transgenic and wild-type plants were grown as control to compare plant growth and flower coloration. PCR analysis showed that the transgene was integrated into the transgenic plant genome and RT-PCR analysis demonstrated the ectopic expression of the VbDFR transgene (Figure 5b). Obvious flower color changes were observed in VbDFR transgenic plants. The red pigmentation of VbDFR transgenic petals was deeper than that of control petals (Figure 5a). Further quantification using methanol: HCl extraction was carried out on a UV spectrophotometer. Absorbance values recorded at 530 nm showed that the contents of anthocyanins are significantly higher in VbDFR transgenic flowers than in control ones (Figure 5c). These demonstrated that the overexpression of VbDFR increased anthocyanin biosynthesis in transgenic flowers.

To further characterize effects of the VbDFR transgene on anthocyanidin profiles in transgenic flowers, anthocyanin extracts were hydrolyzed using butanol: HCl boiling. The hydrolyzed products were subject to TLC profiling and HPLC analysis. The resulting TLC profiles showed that in addition to a strong spot with the same Rf value as cyanidin, red colors were observed in spots with the same Rf values as pelargonidin and delphinidin (Figure 5d). To further characterize anthocyanidins corresponding to these spots, HPLC analysis were performed to compare them with three authentic standards, cyanidin, pelargonidin, and delphinidin, which have the maximum absorbance value at 524 nm, 515 nm, and 530 nm, respectively. Based on the retention time and UV spectrum properties, cyanidin, pelargonidin, and delphinidin were detected in VbDFR transgenic flower extracts, while only cyanidin was detected from wild-type control flowers (Figure 5e). Moreover, the peak area of cyanidin from the VbDFR transgenic flowers were significantly bigger than that from wild type flowers. We noticed that on the TLC plate, the light pink spot at the same retention time as pelargonidin was obvious, however HPLC only showed a relatively small shouldered peak (Figure 5e-2). This likely resulted from the overlapping of an unidentified anthocyanidin and pelargonidin on the TLC plate, two of which were separated by HPLC. These results further demonstrated that the overexpression of VbDFR in tobacco not only increased cyanidin contents, but also produced two new tobacco anthocyanidins.

## 3. Discussion

The study of more *DFR* homologs from different *Vitis* species is necessary in order to appropriately understand this key gene’s function in the biosynthesis of plant flavonoids for grape agriculture. Since the *VvDFR* cDNA sequence was cloned from *V. vinifera* two decades ago [31], to our knowledge, no *DFR* homologs have been characterized from other *Vitis* species. To date, *VvDFR* is the only *Vitis* DFR homolog that has been appropriately characterized to understand the step from dihydroflavonols to leucoanthocyanidins in the biosynthetic pathways of anthocyanins and proanthocyanidins in grape berries [32,33,34,35,36]. Different transcriptional studies have showed that the expression of *VvDFR* is regulated by different factors. High temperature during night time was found to inhibit the expression of *VvDFR* [34]. Light was found to induce the activation of the DFR promoter [32]. The synthetic auxin, 2,4-dichlorophynoxyacetic acid, was found to inhibit the expression of *VvDFR* in anthocyanin producing cells, which abscisic acid was shown to upregulate the expression of *VvDFR* [37]. Other factors such as calcium and sucrose were also demonstrated to induce the expression of *VvDFR* [32]. A crystal structural study has characterized the NADPH-binding Rossmann domain at the N-terminus and substrate-binding specificity in the variable C-terminus [36]. The amino acid region from 131–15 has been characterized to be the substrate binding site. In particular, the Asn or Asp residue at position 133 has been characterized to associate with substrate recognition although the variant N133D may not be solely specific to recognize the three hydroxylation patterns in the B-ring of dihydroflavonols (Figure 1) [36]. In summary, on the one hand, these data provide useful information for understanding the regulation of biosynthesis of anthocyanins and proanthocyanidins in grape berries in the field. On the other hand, the function of *VvDFR in planta*, such as in transgenic plants, still remains for further investigation. The present study characterizes the function of *VbDFR*, a new homolog, to understand the biosynthesis of plant flavonoids in the *Vitis* species. It is interesting that VbDFR and VvDFR only have three different amino acids, revealing the high conservation of sequences. Accordingly, as we expected, an in vitro assay showed that the recombinant VbDFR used DHK and DHQ as substrates to produce their corresponding leucopelargonidin and leucocyanidin (Figure 3d,e). Although we couldn’t find DHM for a substrate assay, *VbDFR* transgenic but not control tobacco flowers produced delphinidin, providing evidence that VbDFR uses DHM as substrate. Our confocal analysis showed the subcellular localization of VbDFR in the cytosol (Figure 4), revealing the in vivo catalytic localization in cells. To date, the function of VvDFR has not been characterized in transgenic plants. In our study, we overexpressed *VbDFR* in tobacco plants to understand this *Vitis* DFR’s functions *in planta*. The ectopic expression of *VbDFR* increased anthocyanin production (Figure 5a,d). More importantly, transgenic flowers produced pelargonidin and delphinidin (Figure 5c,e), which were not produced in control flowers, demonstrating that the VbDFR produced new tobacco anthocyanidins. Given that anthocyanidins characterized from *V. vinifera* mainly include cyanidin and delphinidin and their derivatives such as peonidin, petunidin, and malvidin [35], while pelargonidin and pelargonins are uncommonly identified from this crop, our transgenics data provide new information to understand the structural diversity of anthocyanidins in Bellula grape plants and suggest that studying more grape species can enhance the discovery of new anthocyanins for wine and non-alcoholic beverage industries.

## 4. Materials and Methods

### 4.1. Cloning of VbDFR from Young Leaves of V. bellula 

Fifty milligrams of young leaf sample of *V. bellula* were collected and ground into fine powder in liquid nitrogen. Total RNA was extracted using a Plant RNA extract Kit (Sangon, Shanghai, China) and then digested with 1.0 μg DNase to remove genomic DNA contamination. The resulting DNA-free RNA sample was used as a template to synthesize the first strand cDNA with MMLV Reverse transcriptase (Takara, Japan) and oligo (dT)_12_ primer. All steps followed those manufacturers’ protocols.

Based on *V. vinifera DFR* sequences, one pair of primers containing the start and stop codons was designed to amplify its homolog from *V. bellula*, namely *VbDFR*. The forward primer was 5′-ATGGGTTCACAAAGTGAAAC-3′, and the reverse primer was 5′-CTAGGTCTTGCCATCTACAG-3′. Two μL of the 1st strand cDNA was used as template for polymerase chain reaction (PCR) to amplify *VbDFR* cDNA. Ex-Taq polymerase (Takara, Japan) was used for PCR following this manufacturer’s protocol. The thermal cycle was composed of 94 °C 5 min, 30 cycles of 94 °C 45 s, 57 °C 45 s, and 72 °C 45 s. The final extension step was 10 min at 72 °C. The amplified cDNA was separated on an agarose gel by electrophoresis and visualized using EB dye. The band was excised from gel, from which the cDNA fragment was purified using a Qiagen DNA purification kit by following the manufacturer’s protocol. Next, the isolated cDNA was cloned into a T-easy vector (Promega, Madison, WI, USA) to obtain a T-VbDFR plasmid by following the manufacturer’s protocol. The new plasmid was introduced into competent cells of *E. coli* DH5α strain. One single colony was selected for suspension culture. The resulting *E. coli* culture was used to isolate the plasmid with QIAprep Spin Miniprep Kit (Qiagin, Hilden, Germany). The resulting plasmid was used for sequencing. 

### 4.2. Expression of Recombinant VbDFR in E. coli and Purification

A pair of primers was designed to clone *VbDFR* to pET28a (+) (Novagen, Madison, WI, USA), a protein expression vector. The forward primer was 5′-CGGAATTCATGGGTTCACAAAGTGAAACCG-3′, in which the underlined region is an introduced *EcoR* I restriction site. The reverse primer was 5′-CCGCTCGAGTTAGTGATGGTGATGATGGTGGGTCTTGCCATCCTACAGG-3′, in which the underlined CTCGAG region is an introduced *Xhol* restriction site and the other is an introduced His-tag encoding site consisting of 18 nucleotides. Approximately 1.0 ng of the T-VbDFR plasmid was used as template for PCR, which was carried out using Ex-taq DNA polymerase (Takara, Japan) as described above. The resulting PCR product was purified as described above and then digested with *Xhol* and *EcoR* I (Takara, Japan) by following the manufacturer’s protocol. The digested products were purified via gel purification as described above and then ligated into the pET28a (+) vector, which was also digested by *Xhol* and *EcoR* I. Ligated products were introduced into competent cells of BL21 (DE3) plysS *E. coli* strain, which were streaked on agar-solidified LB medium plate containing 200 mg/L ampicillin. Single positive colonies were selected via PCR-based screening and then used for suspension culture to isolate the plasmid. The resulting plasmid was used for sequencing to obtain a sequence without mutations. The empty vector was also introduced to *E. coli* as a control.

A positive colony was identified and then inoculated to 10 mL autoclaved liquid LB medium supplemented with 200 mg/L ampicillin in a 50 mL tube, which was placed on a rotary shaker at a speed of 250 rpm at 37 °C. After an overnight culture, one mL suspension was inoculated to 100 mL fresh liquid LB containing 200 mg/L ampicillin in a 500 mL Erlenmeyer flask, which was placed on the same shaker at speed of 120 rpm at 37 °C. When the optical density (OD) of suspension culture measured at 600 nm reached 0.7, the incubation temperature was reduced to 30 °C and isopropyl-1-thio-β-d-galactopyranoside (IPTG) was added to cell suspension to a final concentration of 10 mM. Next, cell suspension was continuously cultured 4 h to reach approximately OD_600_ 1.1. Cells were harvested by centrifugation at 6000 rpm 5 min at 4 °C. The supernatant was disposed of to a waste container and the remaining pellet was immediately used for enzyme extraction and purification described below.

Cell pellets were thoroughly suspended in 10.0 mL extraction buffer consisting of 20 mM pH 8.0 Tris-HCl in a 50.0 mL tube. Lysozyme was added into the mixture to a final concentration of 100 µM to treat cells 1 min at room temperature. The mixture was sonicated 30 s on ice. The resulting sticky mixture was configured at 10,000× *g* 10 min. The upper supernatant containing proteins was used for protein purification. The purification of recombinant VbDFR was carried out using a Ni-NTA agarose column. One mL agarose resin (Qiagen, Hilden, Germany) was loaded into a 1 × 10 cm syringe (diameter × height). After the resin had fully sunk to the bottom, it was washed with 10 volumes of extraction buffer (20 mM pH 8.0 Tris-HCl, 10 mM imidazole). Ten mL supernatant cell lysate was loaded onto the top of the resin and flowed through by gravity. The column was then washed using extraction buffer until no proteins were detected from elution. Two mL elution buffer (pH 8.0 20 mM Tris-HCl, 250 mM imidazole) was added to the column to elute recombinant VbDFR. The protein concentration was estimated by Bradford protein assay and the quality of protein was examined on a 10% SDS-PAGE.

### 4.3. Enzymatic Assay

Enzymatic assay was carried out to examine the catalytic activity of the recombinant VbDFR in a 500 μL reaction volume in a 1.5 mL tube. Two commercially available metabolites, taxifolin (dihydroquercetin, DHQ) and (−)-dihydrokaempferol (DHK), were used as substrates. NADPH was used as co-enzyme. Each reaction was composed of 50 mM pH 6 citrate buffer, 20 μg NADPH, 15 μg substrates, and 50 μg purified recombinant VbDFR. The reaction time and temperature were 30 min and 45 °C. The reaction was stopped by addition of 1.0 mL ethyl acetate (EA) and vigorously vortexed. After centrifugation at 10,000 rpm 2 min, the upper EA phase was transferred into a new tube and evaporated in a speedy vacuum at room temperature. The remaining residue was dissolved in 50 μL methanol for HPLC analysis. 

HPLC analysis of VbDFR products was performed on a Shimadzu LC-20AT instrument equipped with an SPD-M20A photodiode array detector (Shimadzu, Japan). Metabolites were separated with a diamonsil C18 reversed phase column and detected at 280 nm. The elution solvent system consisted of 1% phosphate (solvent A) and methanol (solvent B). For taxifolin assay, a linear gradient program was developed to elute metabolites, which was composed of three gradient ratios of B:A, 0–8 min: solvent B from 15% to 60%, 9–15 min: solvent B from 60–15%, and 16–25 min: 15% solvent B at a flow rate 1.5 mL/min. For DHK assay, a different linear gradient program was developed for metabolite separation, which was composed of three gradient ratios of B:A, 0–20 min: solvent B from 15–60%, 21–28 min: solvent B from 60–15%, and 29–35 min: solvent B 15% at a flow rate of 1 mL/min.

### 4.4. GFP Fusion and Transient Expression

The construction of VbDFR-GFP fusion in pBI121 binary vector [1] was carried out with NEBuilder^®^ HiFi DNA Assembly Cloning Kit (NEB, Herts, UK) following the manufacturer’s protocol. In detail, two primer pairs were designed for PCR to amplify VbDFR and GFP cDNA sequences. For VbDFR cDNA amplification, the forward primer was 5′-CTATGACCATGATTACGCCAATGGGTTCACAAAGTGAAACCG-3′, in which the underlined region was overlapped with 20 nucleotides in the 5-terminus of Hind III restrict site in pBI121. The reverse primer was 5′-GCTCCTCGCCCTTCGACATGGTCTTGCCATCTACAGG-3′, in which the stop codon of VbDFR was excluded and the underlined region was overlapped with 19 nucleotides of the 5-terminus of the GFP codon sequence. For *GFP* amplification, the forward primer was 5′-CCTGTAGATGGCAAGACCATGTCGAAGGGCGAGGAGC-3′, in which the underlined region is overlapped with 19 nucleotides in the 3-terminus of *VbDFR* cDNA without stop codon. The reverse primer was 5′-CGATCGGGGAAATTCGAGCTCTACTTGTACAGCTCGTC-3′, in which the underlined region was overlapped with 20 nucleotides of the 3-terminus of sac I restrict site in pBI121. The resulting two DNA fragments were then ligated to Hind III and sac I sites in digested linear pBI121 using DNA Assembly Cloning Kit. The new binary vector was named as pBI121-VbDFR-GFP, in which VbDFR-GFP was controlled by a 35 promoter (Figure 4a). In addition, the GFP gene was introduced into pBI121 vector to create pBI121-GFP construct (Figure 4b) as a positive control. 

These two recombinant vectors were introduced into competent cells of *E. coli* DH5α strain, respectively. *E. coli* cells were spread on agar-solidified LB medium plates supplemented with 50 mg/L kanamycin. One positive clone for each was inoculated to 10 mL liquid LB medium supplemented with 50 mg/L kanamycin in 50 mL tube, which was placed on a rotary shaker at speed of 250 rpm at 37 °C overnight. *E. coli* cultures were harvested by centrifugation of tubes at 4000 rpm 10 min. The resulting *E. coli* pellets were used to extract plasmids using a QIAprep Spin Miniprep Kit. Plasmids were coated with gold particles for transient expression as reported previously [28]. Onion epidermis was pre-cultured on agar-solidified Murashige & Skoog (MS) medium plates overnight at 25 °C, then were bombarded with plasmid-coated gold particles. All epidermises bombarded were incubated on MS medium plate overnight at 25 °C, followed by examination under a confocal laser scanning microscope (Leica TCS SP2, Leica, Germany). The light wavelength was set at 488 nm to observe GFP signal. Cell images and GFP signal were photographed to visualize subcellular localization of protein.

### 4.5. Overexpression of VbDFR in Tobacco

As reported for two *M. trucatula* DFRs previously [1], the ORF of VbDFR was cloned to the pBI121 binary vector by replacing *GUS*. This cloning generated a new recombinant binary vector, pBI121-VbDFR, in which VbDFR is controlled a 35S promoter. Both pBI121-VbDFR and pBI121 vectors were introduced into *Agrobacterium tumefaciens* strain EHA105 for genetic transformation. MS medium, plant hormones used, and all steps of transformation and selection of transgenic plants followed our protocols published previously [1]. Leaves of sterile seedling were used as explants for genetic transformation. VbDFR and vector control transgenic plants were selected using 50 mg/L kanamycin. Transgenic plantlets and control plants were grown in pot soil and placed side by side in green house to develop flowers. Total RNA was isolated from young leaves of transgenic and control plants as described above. RT-PCR were carried out for genotyping with a pair of gene specific primers. The forward primer was 5′-ATGGGTTCACAAAGTGAAAC-3′, and the reverse primer was 5′-CTAGGTCTTGCCATCTACAG-3′. The thermal cycle was composed of 94 °C 5 min, 30 cycles of 94 °C 45 s, 57 °C 45 s, and 72 °C 45 s. The final extension step was 10 min at 72 °C. In addition, *ACTIN* gene was used as the control. Seeds from T0 plants were collected and germinated on MS medium containing 50 mg/L kanamycin. Resistant T1 seedlings were grown in greenhouse to develop flowers for anthocyanin analysis.

### 4.6. Anthocyanin Extraction, Hydrolysis, TLC Assay, and HPLC Analysis

One hundred milligrams of fresh petal tissues were homogenized into fine powder in liquid nitrogen and transferred into a 1.5 mL tube. One mL extraction buffer (0.5% HCl in methanol:water, 1:1) was added into the tube. The powder was thoroughly suspended by vortexing. The tube was placed in the dark 30 min at room temperature, followed by centrifugation at 10,000 rpm 10 min. The supernatant was pipetted into a new tube that contained 0.2 mL chloroform. The tube was vortexed 30 s, followed by centrifugation at 10,000 rpm 2 min. The chloroform phase containing non-polar compounds was pipetted to a waste bottle. This step was repeated once. The resulting upper methanol and water phase was pipetted into a new tube for estimation of anthocyanins at 530 nm on a UV spectrophotometer (UV-2550, Shimadzu, Kyoto, Japan). Then, 50 μL anthocyanins extract was mixed with 950 μL butanol:HCl (95:5, *v*/*v*) in a 1.5 mL tube. The mixture was boiled 1 h, cooled to room temperature, and then evaporated in a speed vacuum. The remaining anthocyanidin residue was suspended in 50 μL of methanol with 0.1% HCl for TLC and HPLC analysis described below. 

TLC assay of anthocyanidins was described previously [18]. In brief, 10 μL of methanol extract of anthocyanidins was loaded onto a cellulose F-200 μM plate. Three authentic standards, pelargonidin chloride, cyanidin chloride, and delphinidin chloride, were also loaded onto TLC plates as positive controls.

The same HPLC instrument and column described above were used to analyze anthocyanidins. The elution solvent system was composed of 0.1% acetic acid (solvent A) and acetonitrile (solvent B). The gradient program composed of different ratios of solvent A to solvent B to elute anthocyanidins. The program consisted of 90:10 to 83:17 (0–5 min), 83:17 to 77:23 (5–10 min), 77:23 to 71:29 (10–15 min), 71:29 to 68:32 (15–20 min), 68:32 to 65:35 (20–25 min), 60:35 (25–39 min), 65:35 to 50:50 (39–45 min), 50:50 to 70:30 (45–50 min), and 70:30 to 90:10 (50–55 min), and then followed by 10 min of column washing. The injection volume and flow rate was 5 μL and 1 mL/min, respectively. Chromatograph was recorded at 530 nm. Three authentic standards, pelargonidin chloride, cyanidin chloride, and delphinidin chloride, were injected as positive controls.
